# Editorial: Diagnostic Approaches for Aspergillus Infections

**DOI:** 10.3389/fmicb.2019.00446

**Published:** 2019-03-19

**Authors:** Helmut J. F. Salzer, Juergen Prattes, Martin Hoenigl

**Affiliations:** ^1^Department of Pulmonary Medicine, Kepler University Hospital, Linz, Austria; ^2^Institute of Nuclear Medicine and Endocrinology, Kepler University Hospital, Linz, Austria; ^3^Section of Infectious Diseases and Tropical Medicine, Medical University of Graz, Graz, Austria; ^4^Division of Infectious Diseases, Department of Medicine, University of California, San Diego, San Diego, CA, United States

**Keywords:** galactomannanan, azole resistance detection, PCR, chronic pulmonary aspergillosis, immunoPET

Infections caused by *Aspergillus* spp. affect immunocompromised patients, patients with certain genetic defects including CARD-9 deficiency (Vaezi et al.,) and patients with pre-existing lung conditions or liver cirrhosis, and are associated with devastating mortality rates (Cornely et al., 2017; Prattes et al., 2017; Hoenigl et al., 2018b). Early and reliable diagnosis and subsequent rapid initiation of appropriate antifungal therapy has shown to improve survival significantly, at least for invasive Aspergillosis (Heldt et al., 2018). However, invasive *Aspergillus* infections progress rapidly and are difficult to diagnose especially at early stages (Hoenigl et al., 2019). Culture based approaches are important for detection of fungal species and resistance testing, however they are limited by low sensitivities—in particular during early phases of infection—and long turnaround time (Eigl et al., 2017). Important advances to the field were brought by the introduction of non-cultural diagnostic tests for aspergillosis in blood and bronchoalveolar lavage fluid, including, galactomannan antigen testing, PCR, and 1,3-ß-D-glucan testing in patients at risk (Eigl et al., 2017; Heldt et al., 2018; Prattes et al., 2018; Jenks et al., 2019b). Complicating is the fact that performance of these tests may vary not only by fungal disease, but also by risk group (e.g., neutropenic patients vs. non-neutropenic patients, neonates vs. adults, antimould prophylaxis vs. no antimould prophylaxis) (Eigl et al., 2015).

The current Research Topic includes in total 19 high quality manuscripts, ranging from reviews of current state of the art of treatment of aspergillosis in solid organ transplant recipients (Herrera and Husain), the pediatric population (Lehrnbecher et al.), and the veterinary setting (Elad and Segal), to a variety of original articles focusing on new diagnostics of invasive and chronic forms of aspergillosis, including detection of azole resistance.

Importantly, several new diagnostic approaches for diagnosis of invasive aspergillosis have been studied within the last years (e.g., novel *Aspergillus* PCR (Rath and Steinmann) assays for which results were shown to be quantitatively correlated to galactomannan levels in one study of this Research Topic (Alanio et al.), the *Aspergillus* specific lateral flow assay (Hoenigl et al., 2018a; Jenks et al., 2019a,b; Salzer et al.), Triacetylfusarinine C (Skriba et al.; Hoenigl et al., 2019), Bis(methylthio)gliotoxin (Vidal-Garcia et al.), PET imaging studies (Thornton), interleukins (Goncalves et al.; Heldt et al., 2017, 2018). These new diagnostic approaches may overcome the limitations observed with the currently available diagnostic tools, like e.g., decreased sensitivities under antifungal prophylaxes/treatment, low specificity or long turn-around times (Hoenigl et al., 2018a, 2019; Jenks et al., 2019b). Our Research Topic includes also a review on serum galactomannan testing in a promising indication other than diagnosis, namely for outcome prediction and treatment stratification (Mercier et al.).

Another focus of this Research Topic is the molecular detection of azole resistance, which is infected by *Aspergillus fumigatus (A. fumigatus)* (Tsitsopoulou et al.), but—even more threatening—as reported in this Research Topic also those infected by *A. terreus* (Zoran et al.), which is non-susceptible to Amphotericin B. Knowledge about epidemiology on *Aspergillus* susceptibility patterns represents a corner stone for appropriate antifungal prophylaxis and treatment as in areas with high rates of environmental azole resistance primarily antifungal treatment with azoles may be reconsidered. Data on environmental azole resistance rates, however, are lacking for many geographic regions. Tsitsopoulou et al. therefore investigated the rate of azole resistant *A. fumigatus* strains in South Wales, UK (Tsitsopoulou et al.). Screening 715 environmental soil and air samples from various regions of South Wales, they found a prevalence of azole resistant *A. fumigatus* strains of 6%. In some areas azole resistance rates were even >10%, including botanic gardens in public parks and a public garden within the grounds of a hospital. Those findings are of concern as susceptible patients may be colonized or infected with resistant *A. fumigatus* strains. Rapid detection of azole resistance in *A. fumigatus* strains in such cases is important for initiation of appropriate antifungal treatment. As *Aspergillus* culture requires up to 7 days followed by antifungal susceptibility testing, molecular assays have been developed for rapid detection of mutations associated with azole resistance among *A. fumigatus*. The performance of two commercially available *Aspergillus* PCR assays including detection of resistance mechanism, the AsperGenius®(PathoNostics, Maastricht, Netherlands) and the MycoGENIE®(Ademtech, Pessac, France) was reviewed by Buil et al. and by Rath and Steinmann. The AsperGenius®represents a multiplex qPCR assay targeting *Aspergillus* DNA, differentiates *A. fumigatus* and *A. terreus* DNA, and detects the most common mutations in the Cyp51A gene associated with azole resistance in *A. fumigatus* (TR_34_/L98H and TR_46_/Y121F/T289A). In addition, the assay is able to detect wildtype (WT) and mutations in Cyp51A DNA simultaneously, enabling to detect a co-infection with a WT and an azole resistant strain. In a prospective multicenter study of bronchoalveolar lavage samples from patients with hematological malignancies the AsperGenius^®^ assay showed a promising diagnostic performance with a sensitivity of 84% and specificity of 80% (Chong et al., 2016). Importantly, detection of azole resistance molecular patterns was associated with treatment failure and higher mortality rates compared to infections with WT. The amount of *Aspergillus* DNA in blood samples is relatively lower compared to respiratory samples, complicating, and reducing the sensitivity of the resistance PCR compared to the *Aspergillus* PCR. Even though, sensitivity was 79% and specificity 100% in serum samples in another retrospective study, no resistance patterns could be observed, probably due to the lower fungal burden (White et al., 2015). The MycoGENIE®assay was superior to the AsperGenius®study in BALF samples in another study (Guegan et al., 2018). In a mixed cohort of hematological and non-hematological patients, sensitivity in of the MycoGENIE®assay was 53.7% in hematological patients, and 75% in non-hematological patients while the sensitivity of the AsperGenius®was 41.5 and 60%, respectively. Compared to the AsperGenius®assay, the MycoGENIE®assay however misses WT probes for the resistance marker. In addition, the TR_46_/Y121F/T289A mutation in the Cyp51A gene is not detected by this assay compared to the AsperGenius®assay. Besides the most common Cyp51A mutations are detected by the AsperGenius®assay, other mutations in this gene may lead to azole resistance. Thus, a group of Germany developed six different in-house PCR assays to detect not only TR34, L98H, Y121F, T289A (all also covered by the AsperGenius®assay) mutations, but also M220 and TR46 mutations (not detected by the AsperGenius®assay) (Postina et al.). The in-house PCR assays resulted positive in 61% of biopsies, 29% in cerebrospinal fluid samples (CSF), 67% in BALF samples, and 100% in isolates. AsperGenius®resulted positive in 47% of biopsies, 42% in CSF samples, 58% in BALF samples, and 100% in isolates. Interestingly, the in-house assays detected more Cyp51A mutations compared to the AsperGenius®assay (17 vs. 10). Nevertheless, in-house assays had a significantly longer hands on time compared to the more time saving AsperGenius®assay, currently limiting the use in daily clinical routine.

*Aspergillus* infections and colonization is also causing disease progression and limitation of live quality in patients with cystic fibrosis as *Aspergillus* may lead to severe asthma or allergic bronchopulmonary aspergillosis in up to 15% of these patients. One article of this Research Topic evaluated the performance of two in-house PCR assays as well as the performance of the AsperGenius®and the MycoGENIE®assay for *Aspergillus* detection and detection of azole resistance in sputum samples from cystic fibrosis patients (Guegan et al., 2018). They found comparable performance for all four assays. Of note, a large number of culture negative samples turned out positive with the PCR assays indicating a higher sensitivity within these assays. Azole resistance was present in five cultured isolates recovered from patients with long term azole treatment, of which three displayed mutations in the Cyp51A gene.

In contrast to the invasive form chronic pulmonary aspergillosis (CPA) is usually seen in immunocompetent or mildly immunocompromised patients with underlying respiratory diseases (Salzer et al., 2017). Disease severity and progression is highly variable with a 5-year fatality rate between 40 and 60% (Lowes et al., 2017). Estimated three million people are suffering from CPA globally, but precise epidemiological data are lacking (Godet et al., 2018). Reasons include lack of evidence, but also lack of awareness on the disease itself and the challenge to establish the diagnosis. There is no single test or biomarker that allows CPA diagnosis so far. It needs a combination of clinical, radiological, and mycological characteristics (Denning et al., 2016). It is crucial to be aware of recent advances to apply diagnostic methods and interpret test results correctly.

Takazono and Izumikawa reviewed recent advances in diagnostic methods and proposed an algorithm for the diagnosis of CPA (Takazono and Izumikawa). First, it is important to identify patients at risk including immunocompetent or mildly immunocompromised patients with any underlying respiratory disorder, unspecific symptoms (e.g., fever, cough), inflammatory markers, and radiological deterioration. Second, *Mycobacterium* infections should be excluded. Third, an *Aspergillus* IgG antibody assay (EIA) should be performed in blood. If positive, an optional bronchoscopy should be considered offering further diagnostic methods including histopathology/cytology, fungal culture for drug sensitivity testing, galactomannan (GM), 1,3-ß-D-glucan, PCR, and PCR of azole resistant related gene (e.g., AsperGenius). If *Aspergillus* IgG antibody test result is negative, a bronchoscopy should be performed including further diagnostic methods. If all diagnostic tests from bronchoscopy and the *Aspergillus* IgG antibody are negative, differential diagnosis should be reconsidered, and patients should be followed up. Fourth, patients at risk with CPA typical radiological findings and symptoms with a positive *Aspergillus* IgG test result and/or a positive test result from bronchoscopy with proven mycological evidence should be treated primarily with azoles for at least 6 months.

Radiological presentation is a key diagnostic criterion and most often the first indication of CPA. If available a computed tomography (CT) scan of the chest should be performed. However, different technologies such as [^18^F] fluorodeoxyglucose positron emission tomography ([18F]FDG-PET) CT scan or molecular imaging using antibody-guided PET/magnetic resonance imaging (immunoPET/MRI) are promising new techniques (Thornton). So far, most data are related to IA, but the principal could be used for CPA too.

Most tests used to prove mycological evidence in CPA patients were originally developed for the diagnosis of IA. GM testing from BAL is frequently used in CPA patients with a reported sensitivity and specificity between 78–92 and 76–90%, respectively using an cut-off of 0.5 optical density index (ODI). The most recent study by Salzer et al. showed lower rates of sensitivity of GM testing from BALF with 41 and 30% with a cut off level of 0.5 ODI and 1.0 ODI, respectively (Salzer et al.). However, the specificity was high with 100%. Comparable results were shown for the first time in CPA patients for the newly formatted CE marked *Aspergillus* lateral flow device (LFD). Previous studies included a higher proportion of subacute invasive aspergillosis (SAIA) patients (formally chronic necrotizing or semi-invasive pulmonary aspergillosis), which is a plausible explanation for higher sensitivity rates since SAIA is in fact an invasive form of the disease and very similar to IA. This study has direct implications for clinical routine since GM testing and LFD show an insufficient performance for diagnosing CPA, but it they can contribute to the diagnostic work-up by excluding invasive disease.

Any diagnostic test to proof mycological evidence needs to be interpreted in the clinical and radiological context since direct or indirect *Aspergillus* detection can reflect colonization, allergic- or infectious disease. Barac et al. conducted a prospective cohort study including 75 patients with ABPA with the aim to clarify if allergic bronchopulmonary aspergillosis (ABPA) and allergic fungal rhinosinusitis (AFRS) could be considered as a common disease entity (Barac et al.). AFRS was confirmed in 80% of patients with ABPA. Therefore, specialists have to consider both presentations in their clinical management.

Collectively, the studies described in original research and review articles in this topic describe recent advances and provide optimism for the future of diagnosis of Aspergillus infections. We hope these articles will stimulate further research with the ultimate goal of improving outcomes for patients with Aspergillus disease.

## Author Contributions

All authors listed have made a substantial, direct and intellectual contribution to the work, and approved it for publication.

### Conflict of Interest Statement

HS received speakers honoraria from Chiesi and a research grant from Gilead. JP received consulting fees from Gilead. MH received research funding from Gilead.
